# Circulation of *Dirofilaria repens* and *Dirofilaria immitis* in Moldova

**DOI:** 10.1186/s13071-016-1916-4

**Published:** 2016-12-03

**Authors:** Tatiana Șuleșco, Heidrun von Thien, Lidia Toderaș, Ion Toderaș, Renke Lühken, Egbert Tannich

**Affiliations:** 1 Institute of Zoology, Academy of Sciences of Moldova, Chisinau, Moldova; 2 Bernhard Nocht Institute for Tropical Medicine, WHO Collaborating Centre for Arbovirus and Hemorrhagic Fever Reference and Research, Hamburg, Germany; 3 German Centre for Infection Research (DZIF), partner site Hamburg-Luebeck-Borstel, Hamburg, Germany

**Keywords:** *Dirofilaria repens*, *Dirofilaria immitis*, Vectors, *Dirofilaria* development units

## Abstract

**Background:**

Over the last two decades, a significant spread of dirofilariasis has been observed in eastern and central Europe. However, data on the circulation of *Dirofilaria* spp. in Moldova were absent although direct neighbor states reported high incidence rates of human dirofilariasis.

**Methods:**

Daily mean temperature data were used to calculate *Dirofilaria* spp. development units, which were used to estimate the potential for complete extrinsic development in the mosquitoes (= sum of potential *Dirofilaria* spp. transmission days). In addition, 4,481 adult female mosquitoes were collected from 25 trapping sites. From 2010 to 2015, sampling was conducted with Centers for Disease Control miniature light traps, indoor resting mosquito collections as well as human landing catches in urban, rural and natural areas. Mosquitoes were analyzed for the presence of *D. repens* and *D. immitis* DNA using a duplex real-time PCR assay targeting nucleotide differences within the cytochrome *c* oxidase subunit 1 (*D. repens*) and 16S rRNA gene fragment (*D. immitis*).

**Results:**

The average of the yearly sum of potential *Dirofilaria* spp. transmission days between 2010 and 2015 ranged from 90 to 140 days with an increasing gradient from the North to the South of Moldova. Positive mosquito pools for *D. repens* were found countrywide at 13 of the 25 trapping sites and in 17 of the 22 screened mosquito taxa (26.51% of all 347 tested pools), while *D. immitis* was detected only at 4 of the trapping sites (Center and South) in 4 different mosquito species (8.65% of all 347 tested pools). Highest infection rates (EIR) per 100 specimens for both *Dirofilaria* species were found in *An. maculipennis* (*s.l*.) (*D. repens*: EIR = 4.91; *D. immitis*: EIR = 2.01), whereas the most frequent mosquito taxon *Cx. pipiens* (*s.l*.)/*torrentium* had significantly lower infections rates (*D. repens*: EIR = 0.88; *D. immitis*: EIR = 0.47).

**Conclusions:**

The temperature conditions in Moldova are suitable for transmission of *Dirofilaria* spp. within the entire country, which is supported by a wide distribution of *Dirofilaria* spp.-positive mosquitoes with high infection rates. The low number of reported human cases most likely does not reflect the current epidemiological situation of dirofilariasis in Moldova.

**Electronic supplementary material:**

The online version of this article (doi:10.1186/s13071-016-1916-4) contains supplementary material, which is available to authorized users.

## Background

Nematodes of the genus *Dirofilaria* (Spirurida: Onchocercidae) are mosquito-borne parasites, infecting wild and domestic mammals of different orders with canids as predominant definitive host [1]. In Europe, *D. repens* Railliet & Henry, 1911 and *D. immitis* (Leidy, 1856) are the causative agents of dirofilariasis [2]. With few exceptions [3–8], humans are dead-end hosts for the parasites as they usually do not develop to the fertile adult stage, but infections can result in pulmonary and subcutaneous nodules. However, in rare cases, severe clinical manifestations affecting various organs have been reported [3, 9].

Over the last two decades, a significant spread of human *Dirofilaria* spp. infections has been observed in eastern and central Europe, including an increase of human cases [10, 11]. Moreover, autochthonous cases were detected in countries, which were previously regarded as non-endemic: Austria [12], Poland [13], Germany [14], Czech Republic [15] and Belarus [16]). Moldova is bordered to the East and South by the Odessa region of the Ukraine where incidence rates for human dirofilariasis ranged from 2.43 to 3.71 per 100,000 inhabitants between 1997 and 2012 [10]. In the eastern neighbor state Romania, at least 12 autochthonous human cases were detected since 2009 and nearly all cases were reported from southern and eastern parts of the country, close to the border with Moldova [17–19]. However, precise information on the prevalence of *Dirofilaria* spp. in Moldova does not exist. Due to the lack of diagnostic capacities and the low awareness of physicians, human cases of dirofilariasis are usually detected by chance and the few published case reports probably do not reflect the current epidemiological situation in the country. Only five cases of human dirofilariasis have been reported so far. The first documented autochthonous human case has been detected in Hincesti (central Moldova) in 1968 [20]. Three autochthonous human cases of ocular dirofilariasis were reported from Tiraspol (2000), Chisinau (2007) and Bender (2009) in eastern and central Moldova [21, 22]. In 2011, the most recent clinical case of subcutaneous dirofilariasis has been described, but from the case description it is unclear whether the patient resided in Moldova and whether this case was autochthonous or imported [23]. Species identification of the isolated nematodes from subcutaneous or ocular lesions was based on microscopic evaluation of morphological characters only. In addition, there are hardly any studies evaluating *Dirofilaria* spp. infections in the local canine populations and only one recent study reports the presence of *D. immitis* identified by morphology in 24% of examined dogs from central Moldova, indicating circulation of the parasite at least in this part of the country [24].

Due to the lack of a systematic xenomonitoring of dogs, humans or mosquitoes, the prevalence and risk of *Dirofilaria* spp. transmission in Moldova is unknown. Therefore, in a first step, *Dirofilaria* spp. transmission days were calculated on the base of daily mean temperature data to assess the nationwide potential risk of transmission. In addition, a molecular screening of field-collected mosquitoes was performed to confirm circulation of *D. repens* and *D. immitis*, to identify potential mosquito vector species, and to get information about the spatial-temporal distribution of both parasite species. Furthermore, the study was used to compare the *Dirofilaria* spp. screening results between the three trapping methods used, i.e. Centers for Disease Control miniature light traps (CDC traps), indoor resting mosquito collections and human landing catches.

## Methods

### Mosquito sampling and species identification

**Fig. 1:**
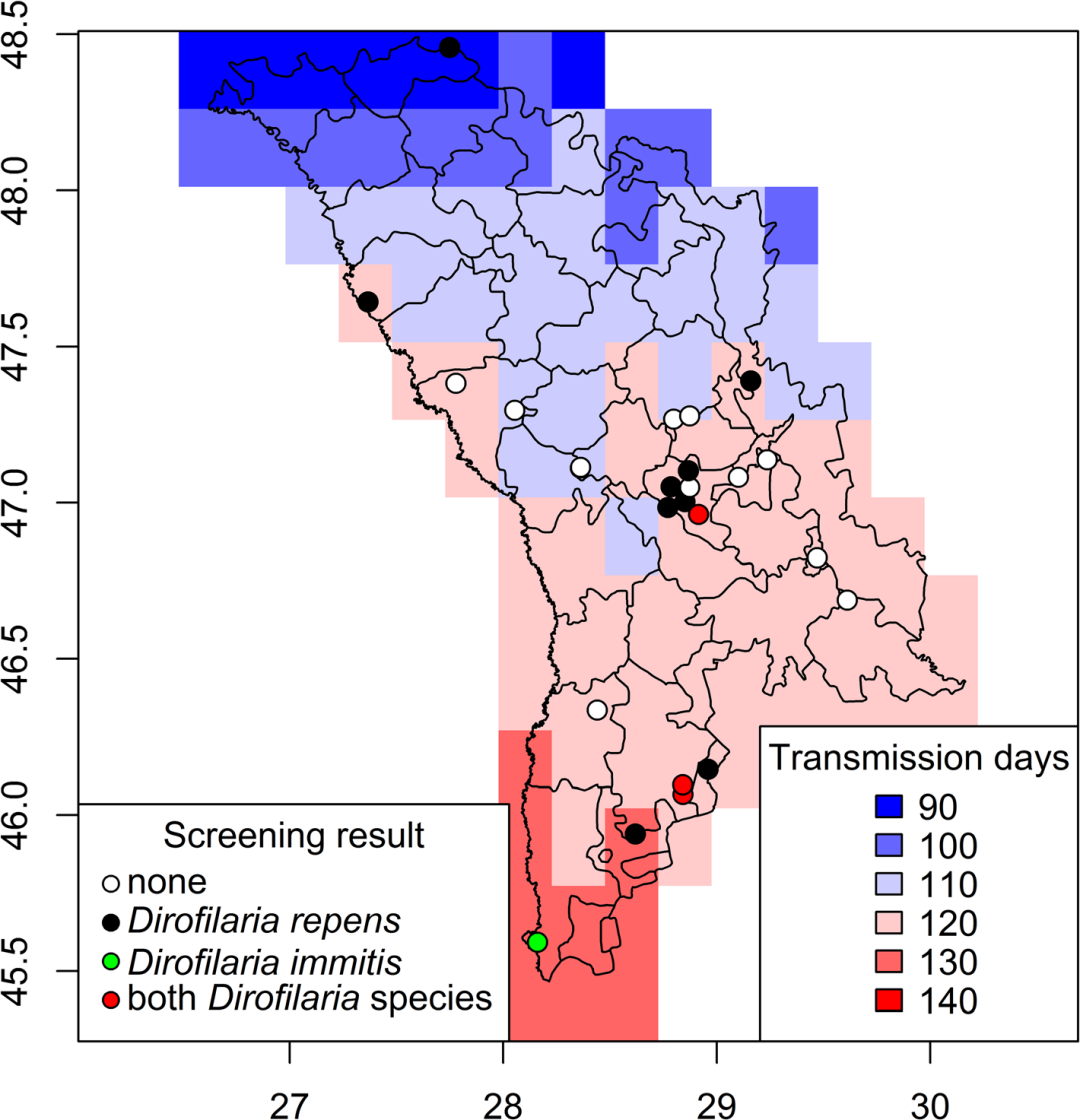
Average of the yearly sum of potential *Dirofilaria* spp. transmission days in Moldova (2010 and 2015) with information on the mosquito sampling sites and *Dirofilaria* spp. screening results

Within a countrywide field survey, adult mosquitoes were collected from 25 trapping sites in Moldova (Fig. 1). Sampling was performed between 2010 and 2015 in urban, rural and natural areas using CDC traps (model 512, John W. Hock Company, Gainesville, Florida, USA), indoor resting mosquito collections with mouth aspirators and human landing catches (see Șuleșco et al. [25] for further methodological details). Furthermore, one site representative for the rural areas in the southern steppe zone in Moldova has been selected for systematic mosquito collections in the years 2014 and 2015 (Ceadir-Lunga, WGS84 coordinates: 46.06549 N, 28.84219E). Between June and October, two CDC traps were operated and regular collections of indoor resting mosquitoes and human landing catches were conducted. Mosquitoes were transported to the laboratory alive and killed in a freezer at -20 °C for approximately 3–10 min. Mosquitoes were identified to species or species complex according to the taxonomic keys published by Schaffner et al. [26] and Becker et al. [27].

### Molecular *Dirofilaria* spp. screening

Female mosquito specimens were screened for *D. repens* and *D. immitis* [16]. Mosquitoes were pooled by sampling site, sampling date and taxon comprising between 1 to 35 specimens per pool (mean = 12.91) and stored in 96% ethanol until further processing. For DNA extraction, mosquitoes were placed in sterile 2 ml reaction tubes and 1 ml of cell culture medium (high-glucose Dulbecco’s modified Eagle’s medium (Sigma-Aldrich, St. Louis, USA) with 10% heat-inactivated fetal bovine serum, 100 U/ml penicillin, 100 μg/ml streptomycin, and 2.5 μg/ml amphotericin B) and 2 stainless steel beads with a diameter of 5.0 mm were added for homogenization in a TissueLyser (Qiagen, Hilden, Germany) at 50 oscillation/s for 2 min. The suspensions were clarified by centrifugation (13,000× *g* for 5 min), and the supernatant was used for DNA extraction with the MagMAX™ Pathogen RNA/DNA Kit using the MagMAX™ Express-96 Deep Well Magnetic Particle Processor (Thermo Fisher Scientific Inc., Waltham, USA) according to the manufacturer’s protocol. The extracted DNA of each sample was analyzed by quantitative real-time PCR (qPCR) assays for the detection of *D. repens* or *D. immitis* DNA. A cytochrome *c* oxidase subunit 1 gene fragment of *D. repens* was amplified using the primers RepF (5′-GAG ATG GCG TTT CCT CGT G-3′) and RepR (5′-GAC CAT CAA CAC TTA AAG-3′) and the probe RepT (5′-JOE-GTT GCT TTG TTA ATG GTT TAT C-BHQ1-3′; JOE = 6-carboxy-4′,5′-dichloro-2′,7′-dimethoxyfluorescein, BHQ1 = black hole quencher 1). For *D. immitis*, a 16S rRNA gene fragment was amplified using the primers ImmF (5′-CTA TAT GTT ACC TTA ATT GG-3′) and ImmR (5′-CTT AAC CAT TAT CTT AGA TCA G-3′) and the probe ImmT (5′-ROX-GTA GCT AGT AAG TTT ACC TTG-BHQ2-3′; ROX = 6-carboxy-X-rhodamine, BHQ2 = black hole quencher 2). The PCRs were performed with the Rotor-Gene™ 6000 real-time PCR machine (Corbett Research, Sydney, Australia). The reaction mixture (20 μl) contained 10 μl of 2× HotStartTaq Plus Master Mix Kit (Qiagen, Hilden, Germany), 25 mM MgCl_2_, 16 pmol RepT or 0.8 pmol ImmT, 1 mg/ml BSA, 4 pmol and 24 pmol of each ImmF / ImmR or RepF / RepR primer pairs, respectively, and 2 μl of extracted DNA (except non-template controls). The thermo profile included an initial denaturation of 15 min at 95 °C followed by 50 cycles consisting of 15 s denaturation at 95 °C, 30 s of annealing at 61 °C and elongation of 30 s at 72 °C. The PCR ended with a final step of 30 s at 40 °C. Fluorescence signals were measured during each extension phase of the PCR reactions and finally analyzed with the Rotor-Gene™ 6000 software version 6.1.8.1. (Corbett Research, Sydney, Australia). PCR amplicons with positive signals in the qPCR were subjected to DNA sequencing on both strands using the same sets of primers that were used in the qPCR. Sequences were edited and aligned using MacVector software version 14.5 (MacVector, Inc., Cambridge, United Kingdom). Resulting sequences were compared with sequences available in the GenBank database.

### Statistical analyses

Data analysis and visualization was conducted with the program R [28] using the packages *ggplot2* [29], *plyr* [30], *maptools* [31], *raster* [32] and *rgeos* [33]. With the same calculation method used in other studies [34], *Dirofilaria* spp. development units (DDUs) were calculated on the base of daily mean temperature data on a 0.25° regular latitude-longitude grid downloaded from http://www.ecad.eu/ [35]. For each grid cell, the daily sums of temperature degrees above a 14 °C threshold within 30 preceding days (corresponding to the estimated mosquito life span maximum) were computed on the base of the mean daily temperatures between April 1 and October 15 (potential mosquito activity season). Days with a sum of DDUs larger 130 were considered to allow extrinsic development of infective larvae in the mosquitoes and summed for each grid cell and year (= sum of potential *Dirofilaria* spp. transmission days) and finally averaged over the years of the sampling period (2010–2015).

Estimated infection rates (EIRs) with corresponding 95% confidence intervals (95% CI) were calculated over all analyzed pools per mosquito species and *Dirofilaria* species using the *binGroup* package [36]. Point estimates were calculated with biased-corrected maximum likelihood estimation and confidence intervals were skewness-corrected. Non-overlapping confidence intervals were interpreted as a significant difference.

## Results

The average of the yearly sum of potential *Dirofilaria* spp. transmission days between 2010 and 2015 ranged from 90 to 140 days with an increasing gradient from the North to the South (Fig. 1). *Dirofilaria* spp.**-**infected mosquitoes were found in 14 of the 25 trapping sites analyzed. *Dirofilaria repens* had a wide distribution and occurred in all parts of the country at 13 out of 25 trapping sites (52.00%). In contrast, *D. immitis* was only registered at four trapping sites (16.00%) in central and southern Moldova, but not in the northern part of the country. There were no differences in the average sum of potential *Dirofilaria* spp. transmission days between sampling sites that were positive for *D. immitis* (120.87 ± 7.76) or *D. repens* (121.54 ± 10.09).

In total, the survey analyzed 22 mosquito taxa (4,481 specimens) belonging to six genera (*Aedes*, *Anopheles*, *Coquillettidia*, *Culex*, *Culiseta* and *Uranotaenia*). All of them were screened for the presence of *D. repens* and *D. immitis* DNA (Table 1). *Culex pipiens* (*s.l*.)/*torrentium* was the most frequent species examined (59.43%), followed by *An. maculipennis* (*s.l*.) (21.13%), *Ae. vexans* (7.01%) and *Cx. modestus* (4.53%). A total of 347 mosquito pools was screened and 109 pools (31.41%) tested positive (EIR per 100 specimens = 2.89, 95% CI: 2.41–3.44). Ninety-two pools (26.51%) were positive for *D. repens* (EIR = 2.34, 95% CI: 1.92–2.84) whereas 30 pools (8.65%) were positive for *D. immitis* DNA (EIR = 0.71, 95% CI: 0.49–0.99). In addition, 13 pools (3.75%) were tested positive for both *Dirofilaria* species.

**Table 1:** Mosquito taxa collected in Moldova between 2010 and 2015 with information on the number of screened mosquito specimens, tested pools, *Dirofilaria* spp. screening results and estimated infection rates (EIR) per 100 mosquito specimens with corresponding 95% confidence intervals (95% CI)

Mosquito species	No. of mosquito specimens	No. of pools	No. of positive pools (% of tested pools per species)	No. of *D. repens-*positive pools (% of tested pools per species)	EIR (95% CI)	No. of *D. immitis-*positive pools (% of tested pools per species)	EIR (95% CI)
*Culex pipiens* (*s.l.*)/*torrentium*	2,663	132	32 (24.24)	22 (16.66)	0.88 (0.57–1.30)	12 (9.09)	0.47 (0.25–0.79)
*Anopheles maculipennis* (*s.l*.)	947	62	37 (59.67)	31 (50.00)	4.91 (3.43–6.95)	16 (25.80)	2.01 (1.20–3.20)
*Aedes vexans*	314	33	5 (15.15)	5 (15.15)	1.68 (0.65–3.68)	0 (0)	0 (−)
*Culex modestus*	203	25	6 (24.00)	6 (24.00)	3.26 (1.45–6.60)	0 (0)	0 (−)
*Uranotaenia unguiculata*	119	8	1 (12.50)	1 (12.50)	0.81 (0.05–3.94)	0 (0)	0 (−)
*Aedes annulipes*	51	10	3 (30.00)	3 (30.00)	6.96 (2.06–19.19)	0 (0)	0 (−)
*Culiseta annulata*	38	13	4 (30.76)	4 (30.76)	11.34 (4.02–25.12)	0 (0)	0 (−)
*Aedes caspius*	26	13	6 (46.15)	6 (46.15)	22.64 (11.15–39.32)	0 (0)	0 (−)
*Aedes geniculatus*	26	10	2 (20.00)	2 (20.00)	7.45 (1.47–21.85)	0 (0)	0 (−)
*Aedes sticticus*	24	7	1 (14.28)	1 (14.28)	4.43 (0.26–20.71)	0 (0)	0 (−)
*Coquillettidia richiardii*	19	11	3 (27.27)	3 (27.27)	16.25 (4.64–37.89)	0 (0)	0 (−)
*Aedes cantans*	15	5	2 (40.00)	2 (40.00)	14.84 (2.87–43.95)	0 (0)	0 (−)
*Aedes riparius*	9	4	2 (50.00)	2 (50.00)	31.20 (6.08–84.92)	0 (0)	0 (−)
*Aedes dorsalis*	7	1	0 (0)	0 (0)	0 (−)	0 (0)	0 (−)
*Anopheles plumbeus*	4	2	0 (0)	0 (0)	0 (−)	0 (0)	0 (−)
*Culiseta longiareolata*	4	4	2 (50.00)	2 (50.00)	50.00 (10.55–89.45)	0 (0)	0 (−)
*Aedes cataphylla*	3	1	0 (0)	0 (0)	0 (−)	0 (0)	0 (−)
*Anopheles claviger*	3	1	0 (0)	0 (0)	0 (−)	0 (0)	0 (−)
*Aedes behningi*	2	2	1 (50.00)	0 (0)	0 (−)	1 (50.00)	50.00 (3.26–96.74)
*Anopheles pseudopictus*	2	1	1 (100)	1 (100)	50.00 (0.00–100.00)	1 (100)	50.00 (0.00–100.00)
*Aedes flavescens*	1	1	1 (100)	1 (100)	100.00 (100.00–100.00)	0 (0)	0 (−)
*Aedes cinereus/geminus*	1	1	0 (0)	0 (0)	0 (−)	0 (0)	0 (−)
Total	4,481	347	109 (31.41)	92 (26.51)		30 (8.64)	

*Dirofilaria repens* was detected for 17 of the 22 screened mosquito taxa, while *D. immitis* was only found for 4 mosquito taxa (Table 1). The highest EIRs (mean between 50 and 100) were observed for the species represented in small sample sizes (e.g. *Ae. flavescens*, *An. pseudopictus* or *Cs. longiareolata*), though EIR based on small sample sizes do not accurately represent the true infection rate in the population [37]. For mosquito taxa represented by larger sample sizes (> 100 specimens), highest EIRs for both *Dirofilaria* species were found for *An. maculipennis* (*s.l*.) (*D. repens*: EIR = 4.91, 95% CI: 3.43–6.95; *D. immitis*: EIR = 2.01, 95% CI: 1.20–3.20), whereas the most frequent mosquito taxon *Cx. pipiens* (*s.l*.)/*torrentium* had significantly lower infections rates for both nematode species as indicated by non-overlapping confidence intervals (*D. repens*: EIR = 0.88, 95% CI: 0.57–1.30; *D. immitis*: EIR = 0.47, 95% CI: 0.25–0.79).

**Fig. 2:**
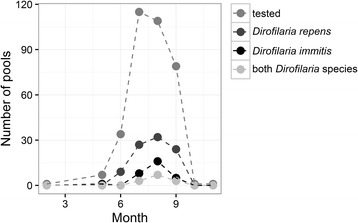
Number of mosquito pools tested per month and the number of pools positive for *D. repens*, *D. immitis* or both *Dirofilaria* species

*Dirofilaria* spp.-positive mosquito pools were detected in each of the 6 study years between May and September (Fig. 2). The highest number of positive pools for both *Dirofilaria* species was recognized in August (44.04% of 109 pools) comprising 29.36% of all tested pools positive for *D. repens* and 14.68% positive for *D. immitis*.

**Fig. 3:**
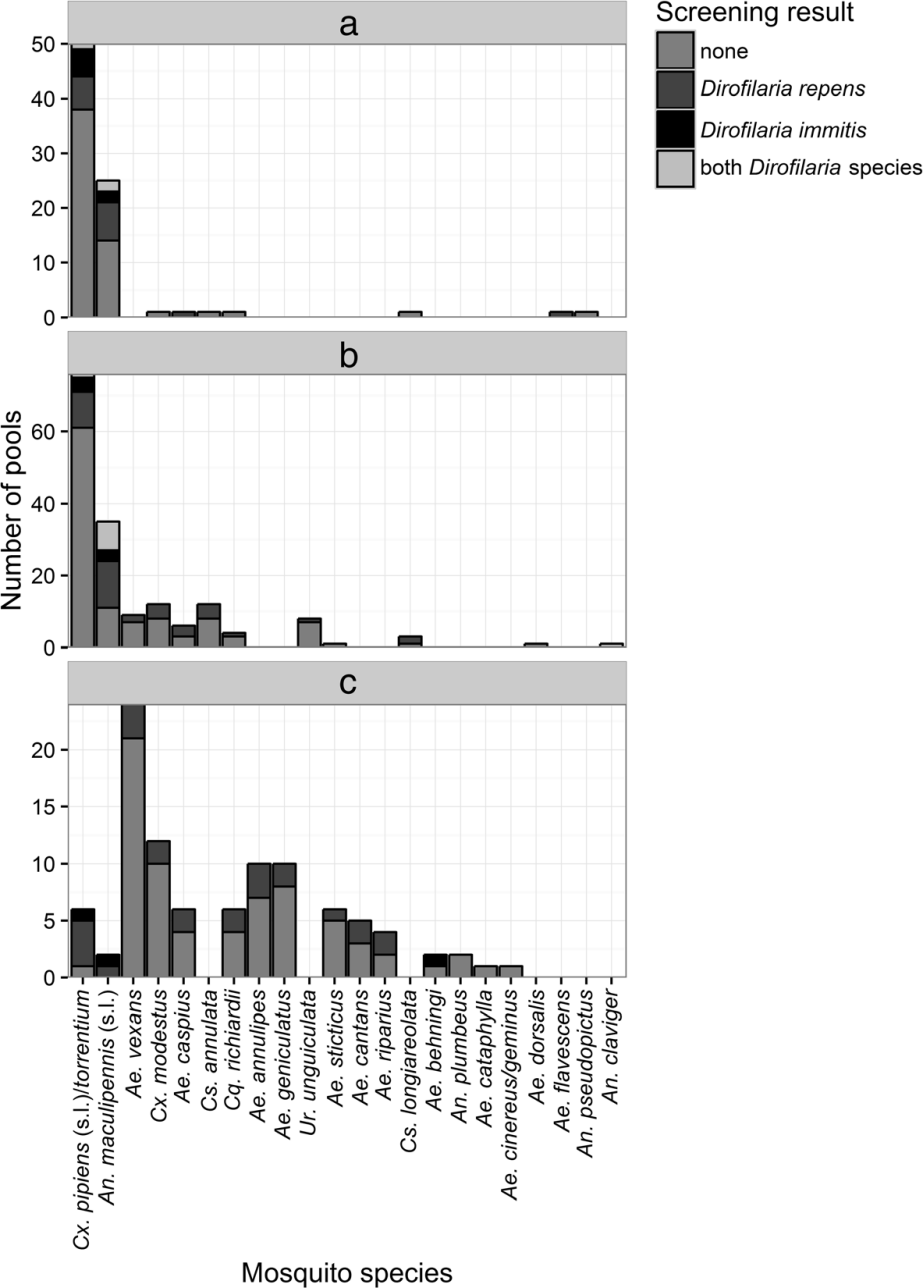
Number of mosquito pools tested per mosquito species and the number of pools positive for *D. repens*, *D. immitis* or both *Dirofilaria* species for the three sampling methods. **a** Indoor resting collection **b** CDC trap **c** Human landing

The number of mosquito specimens and the composition of mosquito species differed between the three sampling methods (Fig. 3). A total of 4,481 mosquito specimens (58.87% of the specimens, 12 mosquito taxa, 54.55% of all collected mosquito taxa) were captured by CDC traps. Mosquito indoor resting collections and human landing catches represented 29.81% (15 mosquito taxa, 68.18%) and 11.31% (9 mosquito taxa, 40.91%) of all mosquito specimens, respectively. The prevalence of both *Dirofilaria* species did not show significant differences between the three trapping methods, but the infection rates for *D. repens* were significantly higher for the mosquitoes sampled with CDC traps and human landing catches compared to the collections of indoor resting mosquitoes (Table 2).

**Table 2:** Prevalence and estimated infection rates (EIR) of both *Dirofilaria* species per 100 mosquito specimens with corresponding 95% confidence intervals (95% CI) for the three sampling methods

	Indoor resting mosquito collections	CDC trap	Human landing
No. of pools tested negative (percentage of all tested pools per sampling method)	57 (69.51)	111 (66.07)	70 (72.16)
No. of pools tested positive for *D. repens* (percentage of all tested pools per sampling method)	15 (18.29)	40 (23.81)	24 (24.74)
No. of pools tested positive for *D. immitis* (percentage of all tested pools per sampling method)	7 (8.54)	7 (4.17)	3 (3.09)
No. of pools tested positive for both *Dirofilaria* species (percentage of all tested pools per sampling method)	3 (3.66)	10 (5.95)	0 (0.00)
EIR for *D. repens* (95% CI)	1.33 (0.82–2.05)	2.98 (2.26–3.86)	3.03 (2.05–4.35)
EIR for *D. immitis* (95% CI)	0.69 (0.35–1.21)	0.89 (0.54–1.40)	0.33 (0.09–0.89)

## Discussion

Various epidemiological studies in Europe report a geographical spread of *Dirofilaria* spp. in eastern and central Europe [10–16]. However, information on the risk of *Dirofilaria* spp. transmission in Moldova is scarce and only few recent studies reported dirofilariasis in dogs [24] or humans [21–23]. Nevertheless, the mean daily temperatures between 2010 and 2015, as analyzed in this study, indicate a potential risk of *Dirofilaria* spp. transmission for the entire country, which is confirmed by the wide distribution of *Dirofilaria* spp.-positive mosquitoes with high infection rates. Thus, this study gives clear molecular evidence for the circulation of *Dirofilaria* spp. and identified several potential mosquito vector species in Moldova, which until recently was considered a non-endemic country.

Of the 36 mosquito species currently known for Moldova [25], 22 mosquito taxa (61.11%) were included in the *Dirofilaria* spp. screening. Our findings suggest that various mosquito species of the genera *Aedes*, *Anopheles*, *Culex* and *Coquillettidia* probably take part in the transmission of *Dirofilaria* spp. in Moldova. Pools of 17 different mosquito taxa (47.22% of the currently known species) were tested positive, which indicates a broad spectrum of potential vector species for *Dirofilaria* spp. transmission in the country. *Dirofilaria* spp. DNA was detected in several mosquito species previously identified as potential vector species during different field-studies in Europe (Table 3). In contrast, the pools of three mosquito species, which were tested positive in previous studies, were negative in this study: *Ae. dorsalis* (*D. repens*: Hungary [38]), *An. claviger* (*D. immitis*: Belarus [16]), *Ae. cinereus*/*geminus* (*D. repens*: Hungary [38]). To the best of our knowledge, *Dirofilaria* spp. were identified for the first time in four species (*Ur. unguiculata*, *Ae. geniculatus*, *Ae. cantans* and *Cs. longioreolata*), which have not been identified as potential vectors in other European countries, where these mosquitoes were studied and dirofilariasis is endemic [38–44]. In addition, this study screened pools of six mosquito species, which were not included in previous studies in Europe. Hereby, pools of the species *Ae. riparius*, *Ae. behningi*, *An. pseudopictus* and *Ae. flavescens* were tested positive whereas pools of *An. plumbeus* and *Ae. cataphylla* tested negative for *Dirofilara* spp. DNA.

**Table 3:** *Dirofilaria* spp.-positive mosquito species in Moldova between 2010 and 2015 previously identified as potential vector species during different field-studies in Europe

Mosquito species	Detected *Dirofilaria* species in Moldavian mosquito species	Countries with detection of *D. repens* in the mosquito species	Countries with detection of *D. immitis* in the mosquito species
*Culex pipiens* (*s.l*.)/*torrentium*	*D. repens* and *D. immitis*	Italy [49], Serbia [39]	Belarus [16], Italy [40, 46, 49, 58], Serbia [39], Turkey [50], Germany [53], Portugal [41], Hungary [63], Spain [42]
*Anopheles maculipennis* (*s.l*.)	*D. repens* and *D. immitis*	Germany [53, 60], Austria [61], Hungary [38]	Italy [40], Portugal [41]
*Aedes vexans*	*D. repens*	Czech Republic [62], Germany [60], Hungary [38], Serbia [39], Slovakia [43, 44]	Italy [58], Turkey [50]
*Culex modestus*	*D. repens*	Hungary [38]	Hungary [63]
*Aedes annulipes*	*D. repens*	Hungary [38]	–
*Culiseta annulata*	*D. repens*	Germany [60]	–
*Aedes caspius*	*D. repens*	–	Italy [58], Hungary [63], Portugal [41], Serbia [39]
*Aedes sticticus*	*D. repens*	Hungary [38], Serbia [39]	
*Coquillettidia richiardii*	*D. repens*	Hungary [38]	Serbia [39], Italy [40]

However, the vector competence for most of these mosquito species remains unknown. Different factors may influence the susceptibility of mosquitoes to *Dirofilara* spp. infections. In addition to damage of microfilariae by species-specific cibarial and pharyngeal armatures, species-specific encapsulation or melanization of the parasite can occur [45]. Furthermore, depending on the microfilarial density in the vertebrate hosts, *Dirofilara* spp. infections may lead to a species-specific increase of mosquito mortality [46, 47], e.g. through invasion of the Malphigian tubule cells [48]. In order to make a definitive assessment of the vector competence of the different mosquito species, some studies in Europe analyzed mosquito heads and abdomens separately to differentiate between infective (potential to transmit the nematode) and infected specimens (only microfilaria in its stomach), but generally did not find significant differences regarding the classification as a vector or non-vector for *Dirofilaria* spp. [40–42, 46, 49–51]. A final assessment of the vector competence requires infection experiments evaluating the susceptibility of the different mosquito species using different microfilarial densities of the vertebrate host under consideration of the impact of *Dirofilaria* spp. infections on the mosquito mortality rate [47].

Nevertheless, in Moldova, members of the taxa *An. maculipennis* (*s.l*.) and *Culex pipiens* (*s.l*.)/*torrentium* are probably the most important *Dirofilaria* spp. vectors. *Anopheles maculipennis* (*s.l*.) was the second most abundant taxon with high infection rates for both *Dirofilaria* species. In contrast, *Culex pipiens* (*s.l*.)/*torrentium* had significantly lower infection rates, but had a three times higher abundance. Members of both species complexes have been previously identified as potential vectors of *Dirofilaria* spp. in infection experiments [52] and in field studies [41, 42, 53]. Furthermore, the collection of infected specimens by human landing collections in this study indicates that members of both species complexes are potential zoonotic vectors of *Dirofilaria* spp. to humans. However, future studies might use recent molecular typing techniques [54–56] to identify the most likely vectors for *Dirofilaria* spp. in these species complexes.

The temporal prevalence of both *Dirofilaria* species followed the phenology of the mosquito abundance and was highest between July and September, which is in concordance with previous reports from Italy [46, 57], while other studies did not find a significant variation through the mosquito season [41, 58]. As discussed previously [46], this pattern is probably predominantly influenced by the local availability of *Dirofilaria* spp. infected dogs and therefore the abundance of the vector species is an important factor driving the spatial-temporal transmission risk of *Dirofilaria* spp. transmission.

*Dirofilaria repens* and *D. immitis* are considered to be transmitted by the same mosquito species [59]. European xenomonitoring studies revealed a variety of *Dirofilaria* spp. infection patterns for the screened mosquitoes, i.e. only *D. immitis* [41, 42, 46, 50], only *D. repens* [38, 60–62] or co-circulation as also observed in Moldova [16, 39, 40, 43, 44, 49, 53, 58, 63]. Nevertheless, the incidence of human dirofilariasis in Europe is significantly higher for infections with *D. repens* than with *D. immitis* [2]. The reasons for the dissimilarities in the epidemiology of both *Dirofilaria* species are unclear. While the pathology in the definitive hosts is well described [2], there is a lack of knowledge about the ecological differences in the intermediate host (e.g. differences in the extrinsic incubation period or host specificity). However, in this study, the number of days allowing the completion of the extrinsic incubation did not differ between the sites positive for *D. repens* or *D. immitis*. One possible explanation of the higher *D. repens* prevalence in Moldova might be the current spread of *D. repens* from eastern Europe to central Europe [10–16]. The most likely explanation for this observation is that *D. repens* infections in dogs are generally asymptomatic, while *D. immitis* infections cause more severe clinical symptoms and, thus, only the latter is recognized and treated to cure the infection [64, 65]. Therefore, *D. repens* can spread unnoticed in the course of increasing dog travel for holidays or relocation [64, 65].

Finally, this study compared three different sampling methods for the monitoring of *Dirofilaria* spp. infected mosquitoes. Compared to the CDC traps, significantly fewer mosquito specimens were collected with human landing catches. Nevertheless, this method helped to identify additional potential *Dirofilaria* spp. vectors. Furthermore, the human landing collections provide important information about the human risk of *Dirofilaria* spp. infection. A large diversity of *Dirofilaria* spp.-infected mosquito species was recognized to feed on humans, underlining the high risk of infection in Moldova. At the same time, the results of the human landing catches support previous studies [66], which indicated that several mosquito species have a much broader host range compared to the classifications found in the literature, e.g. the detection of positive *D. repens* pools for *Cs. longiareolata* and *Ur. unguiculata*, which are expected to predominantly feed on birds or amphibians, respectively [27].

## Conclusion

Although dirofilariasis has been diagnosed in Moldova both, in dogs and humans, solid information on the human risk of infection were missing. The temperature conditions are suitable to allow *Dirofilaria* spp. transmission within the entire country, which is supported by the detection of a wide distribution of *Dirofilaria* spp.-positive mosquitoes with high infection rates in northern, central and southern Moldova. In conclusion, the low number of detected human cases probably does not reflect the current epidemiological situation of dirofilariasis in Moldova and a high prevalence in the local canine populations is expected. Therefore, physicians are advised to consider human subcutaneous and cardiopulmonary dirofilariasis in the differential diagnosis of subcutaneous and pulmonary nodules.

## Additional file

Additional file 1: Table S1. Mosquitoes collected in Moldova between 2010 and 2015 with information on the coordinates of the sampling site, sampling date, mosquito species, pool size and *Dirofilaria* screening results. (XLSX 32 kb)

## Abbreviations

95% CI: 95% confidence interval
CDC traps: Centers for Disease Control miniature light traps
EIR: Estimated infection rate
qPCR: quantitative real-time PCR
